# Supplementary figures and images for: Effects of Drying Methods on Morphological Characteristics, Metabolite Content, and Antioxidant Capacity of Cordyceps sinensis
Source: Foods. 2024 May 24;13(11):1639. doi: 10.3390/foods13111639 (PMC11171906; doi:10.3390/foods13111639)

A

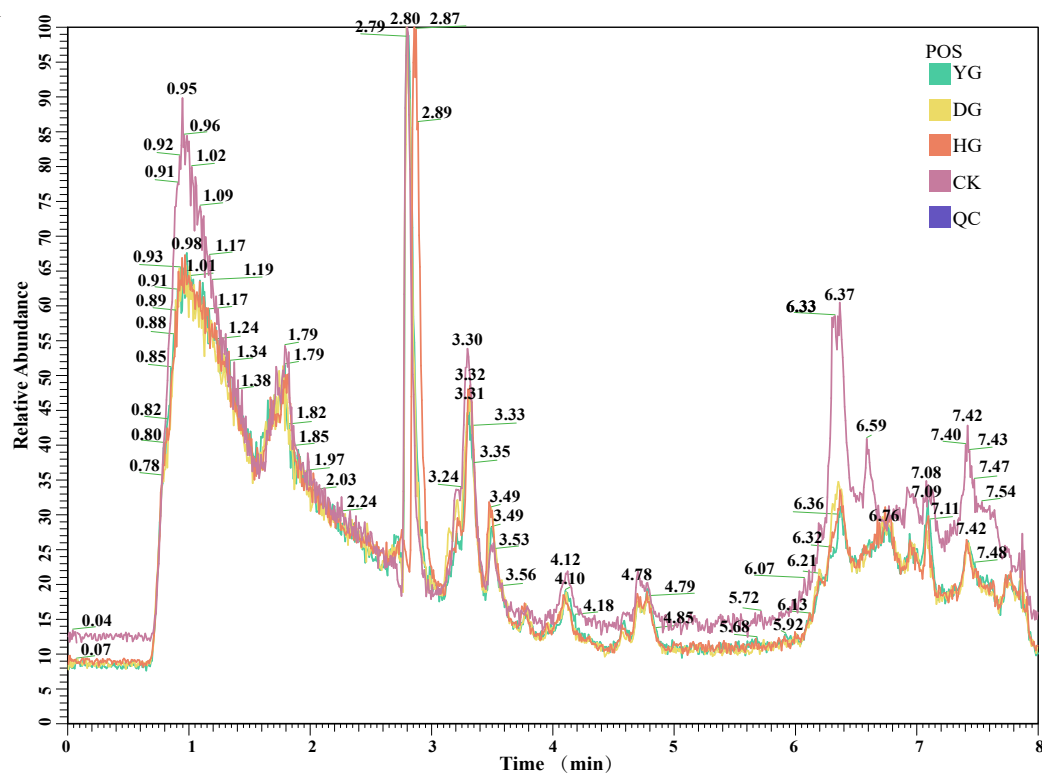

B

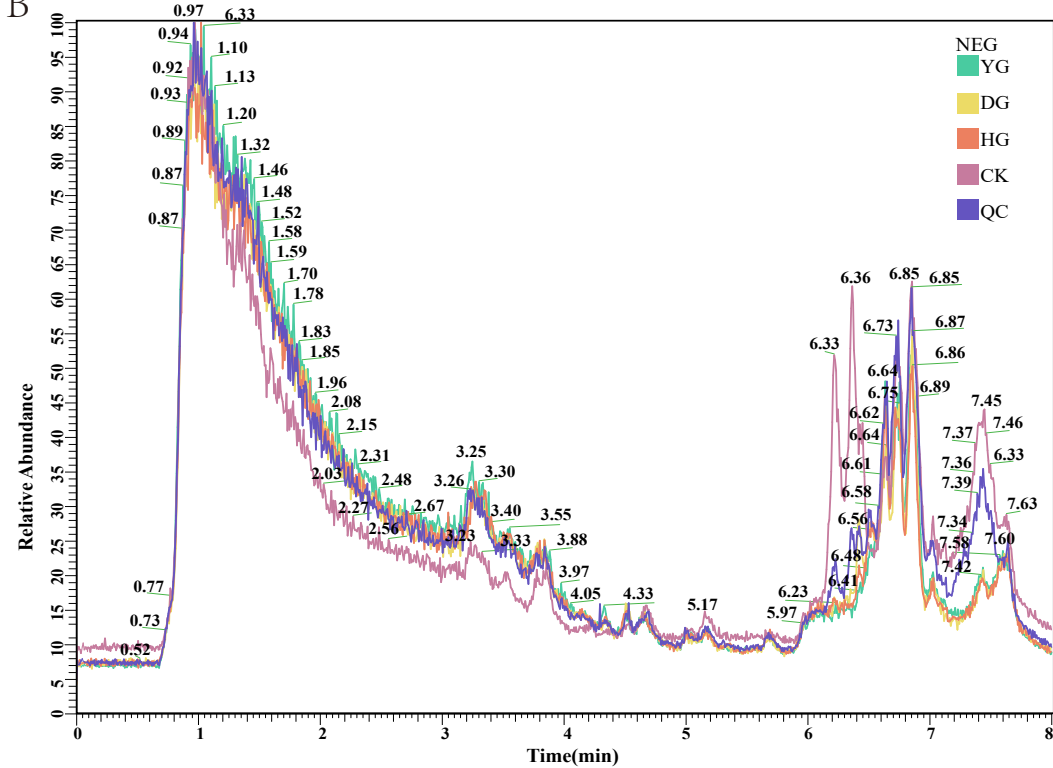

C

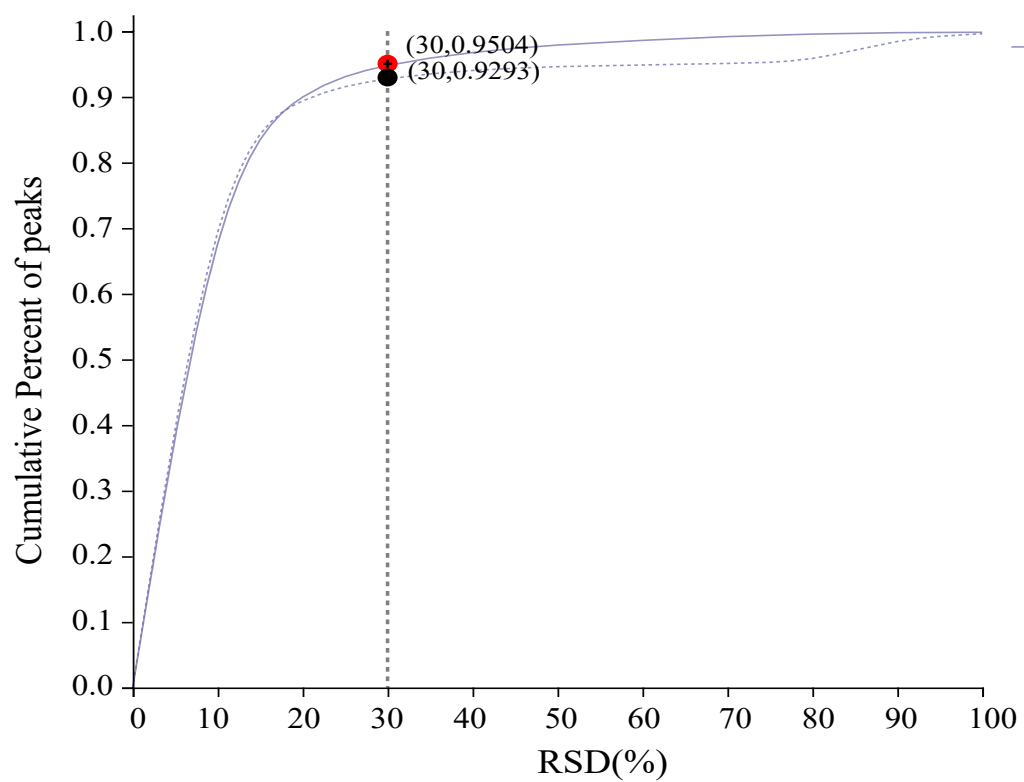

D

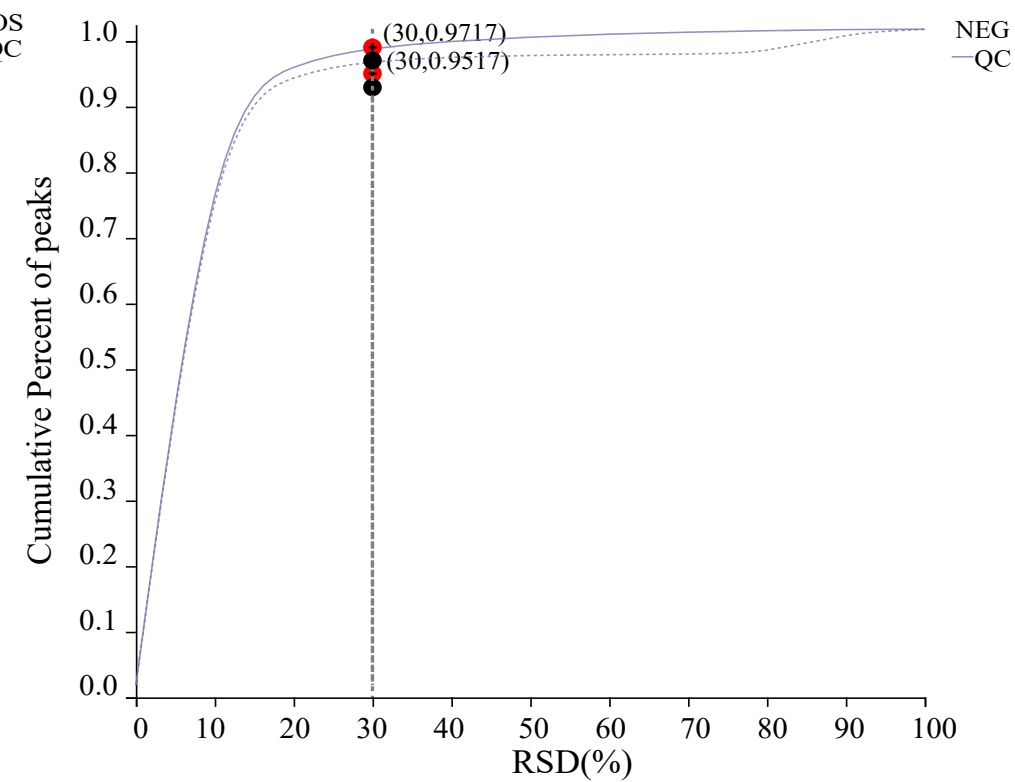

Supplement: Supplementary file 1 [file foods-13-01639-s001.zip › Figure S1.pdf]

A

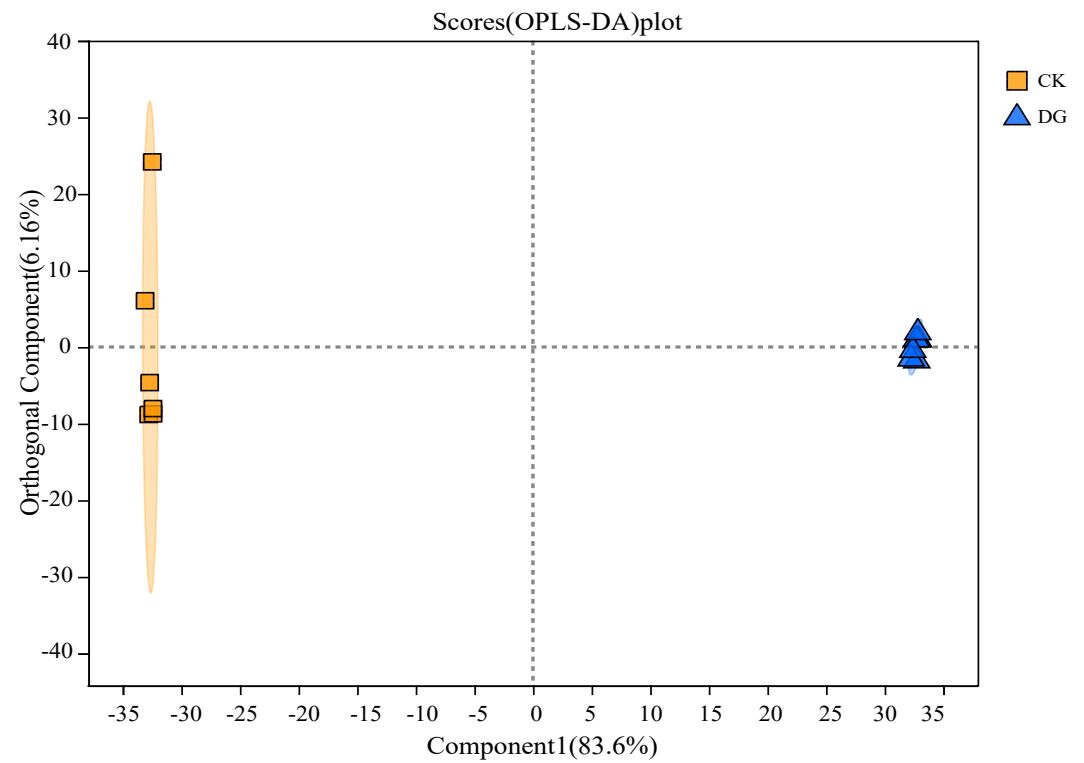

B

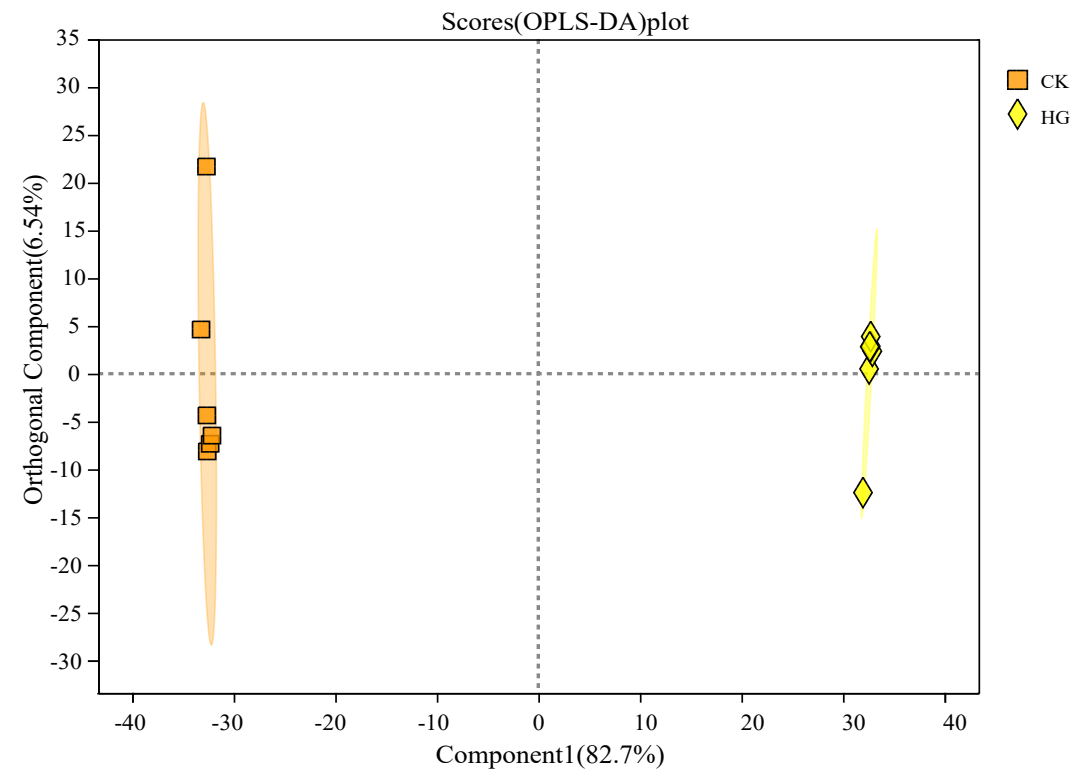

C

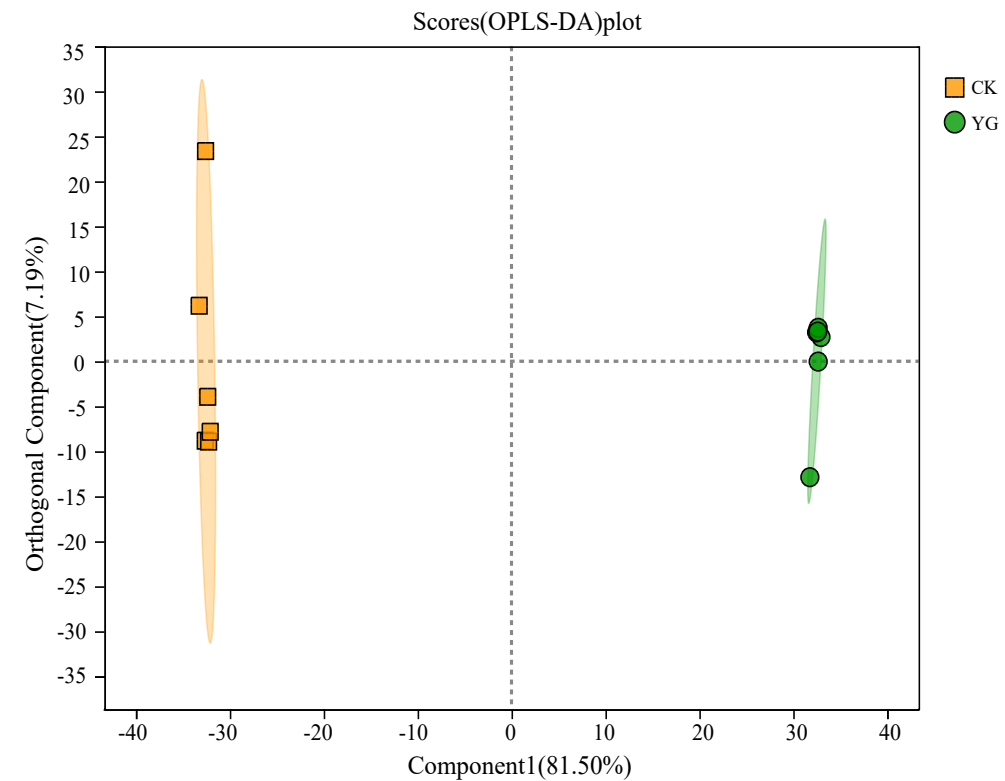

A1

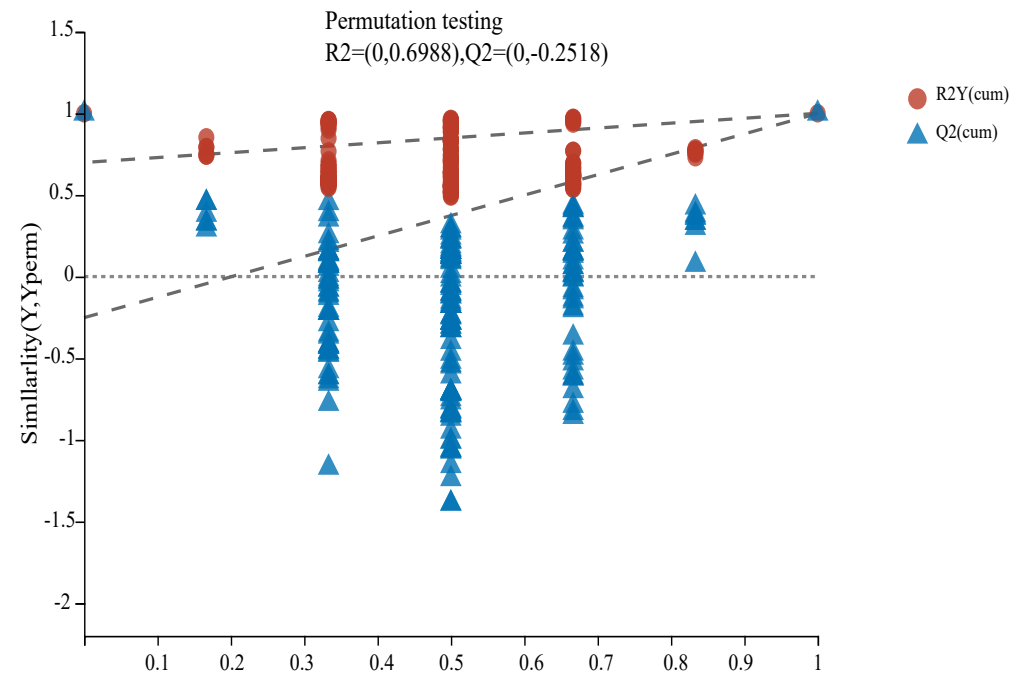

B1

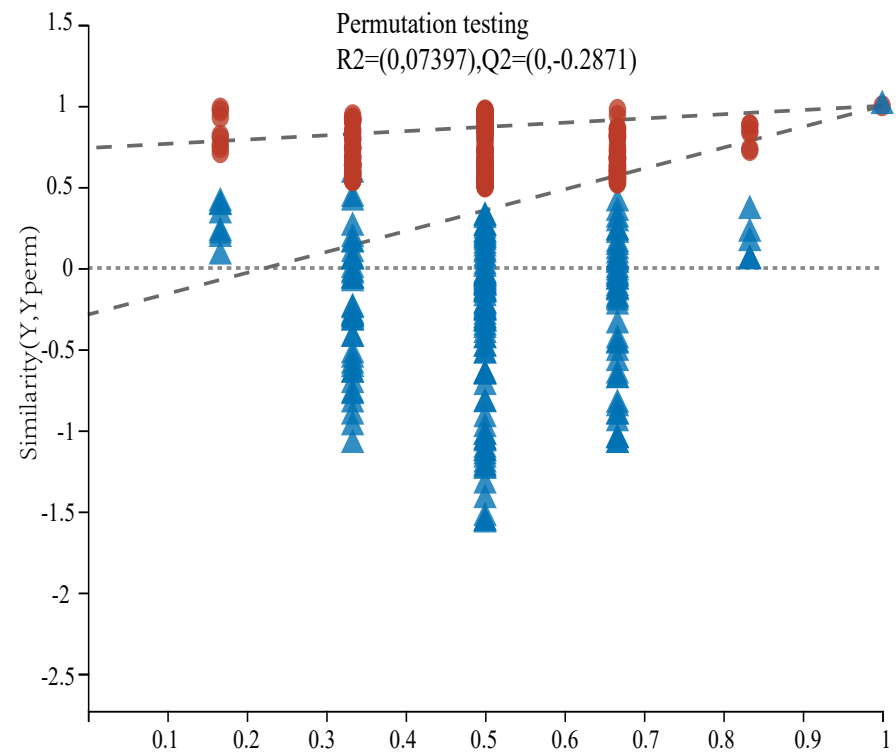

C1

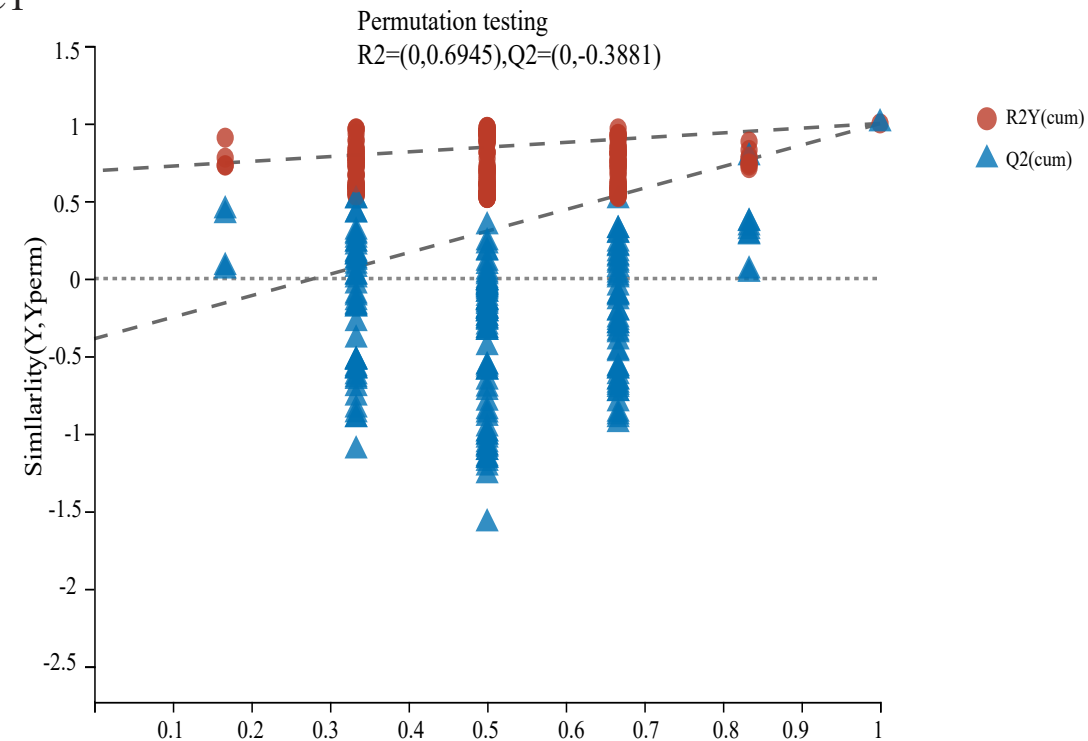

Supplement: Supplementary file 1 [file foods-13-01639-s001.zip › Figure S2.pdf]

A

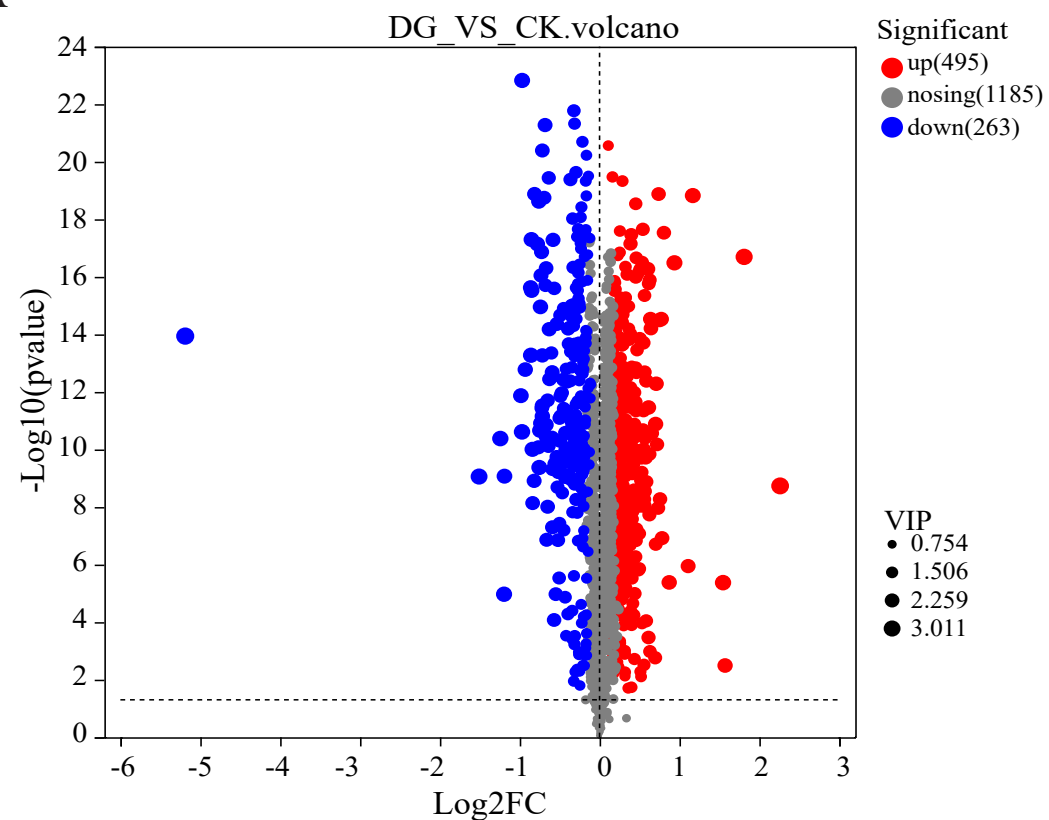

B

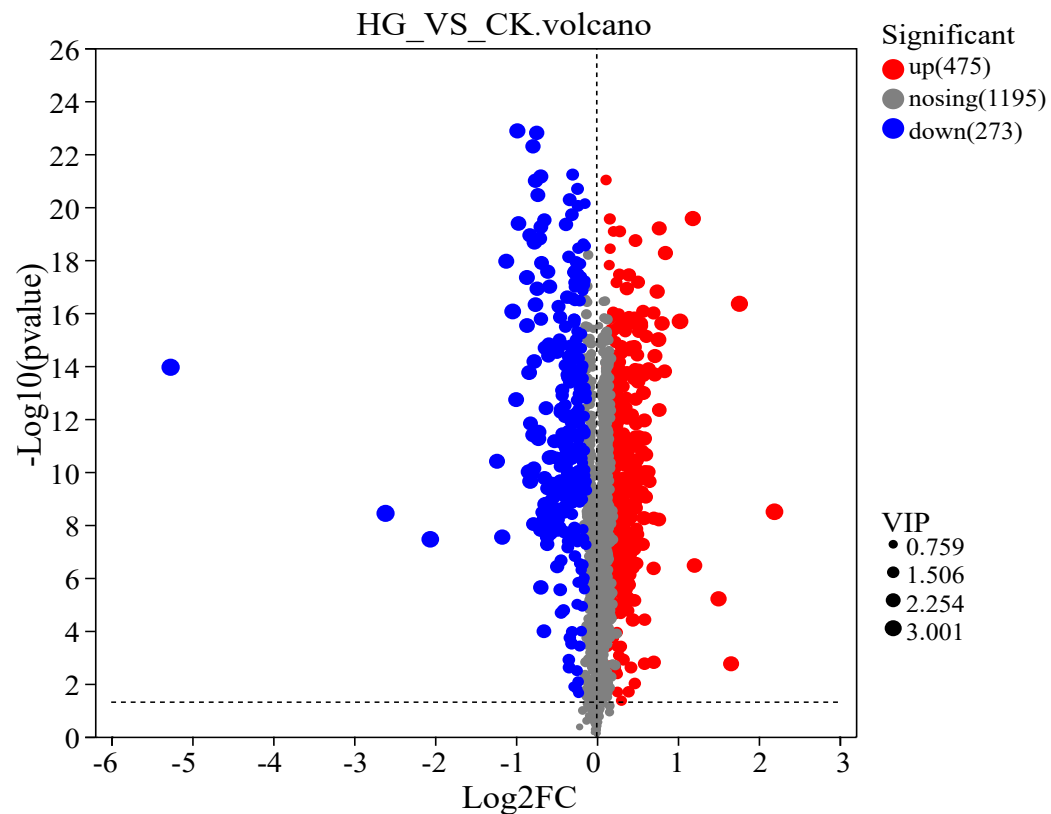

C

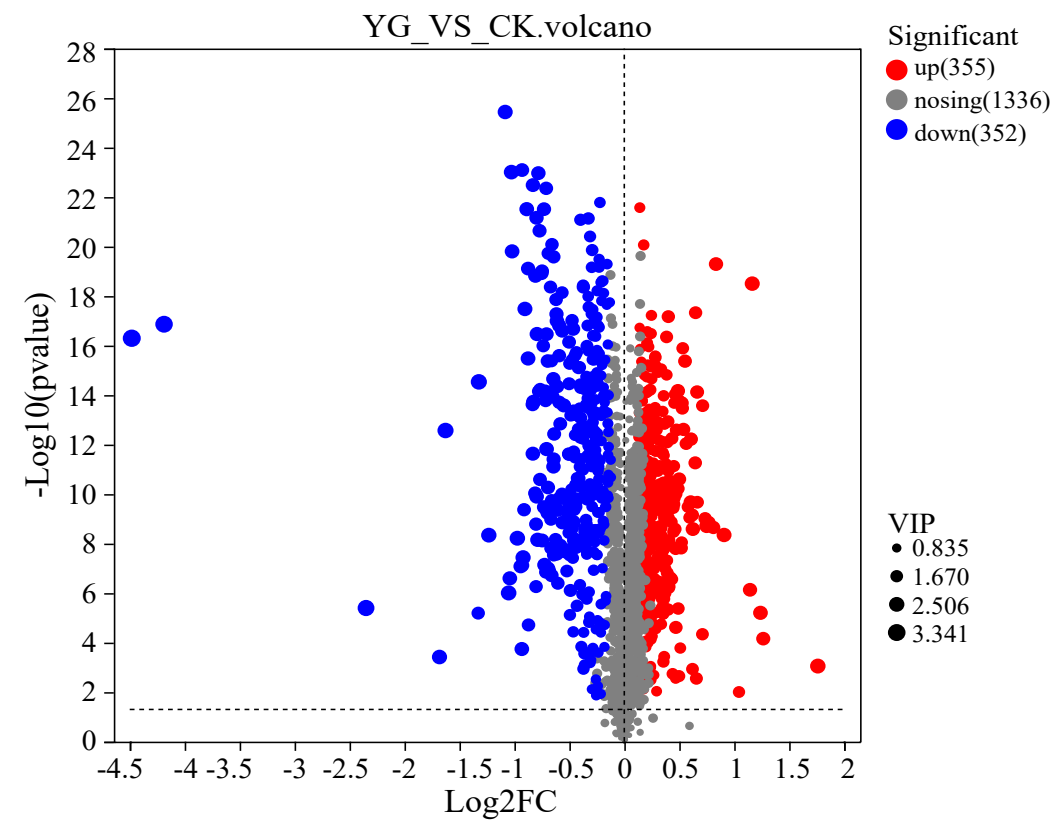

D

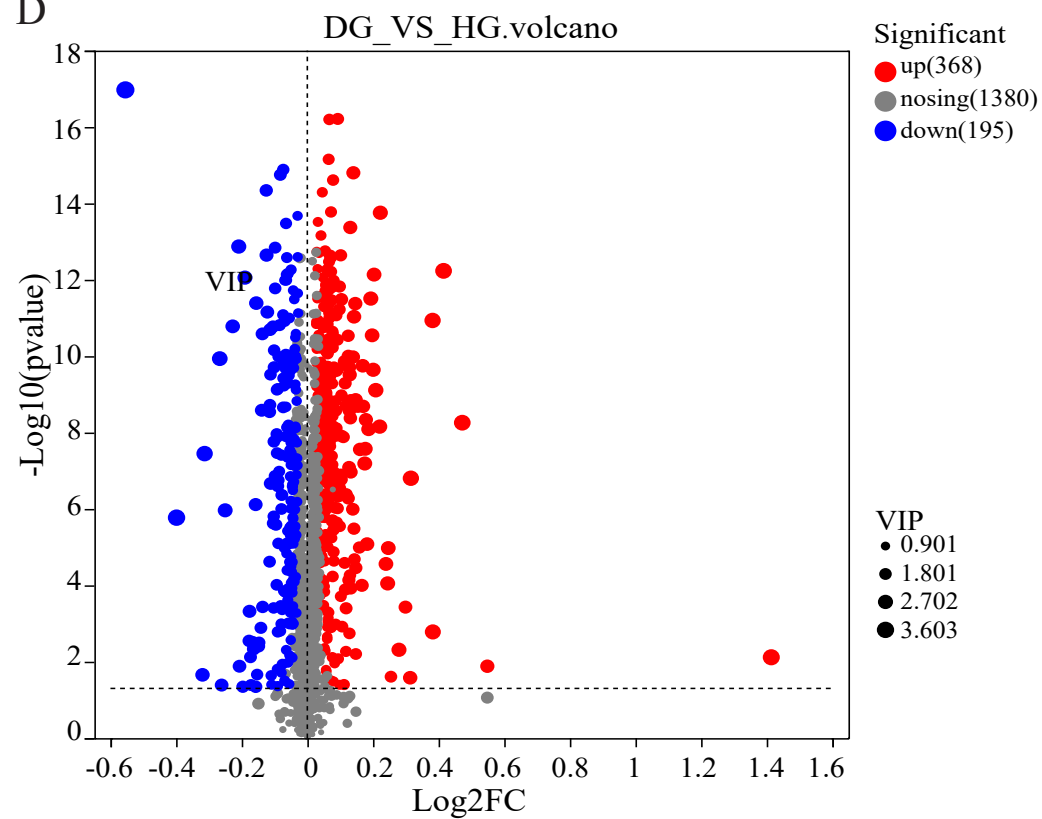

E

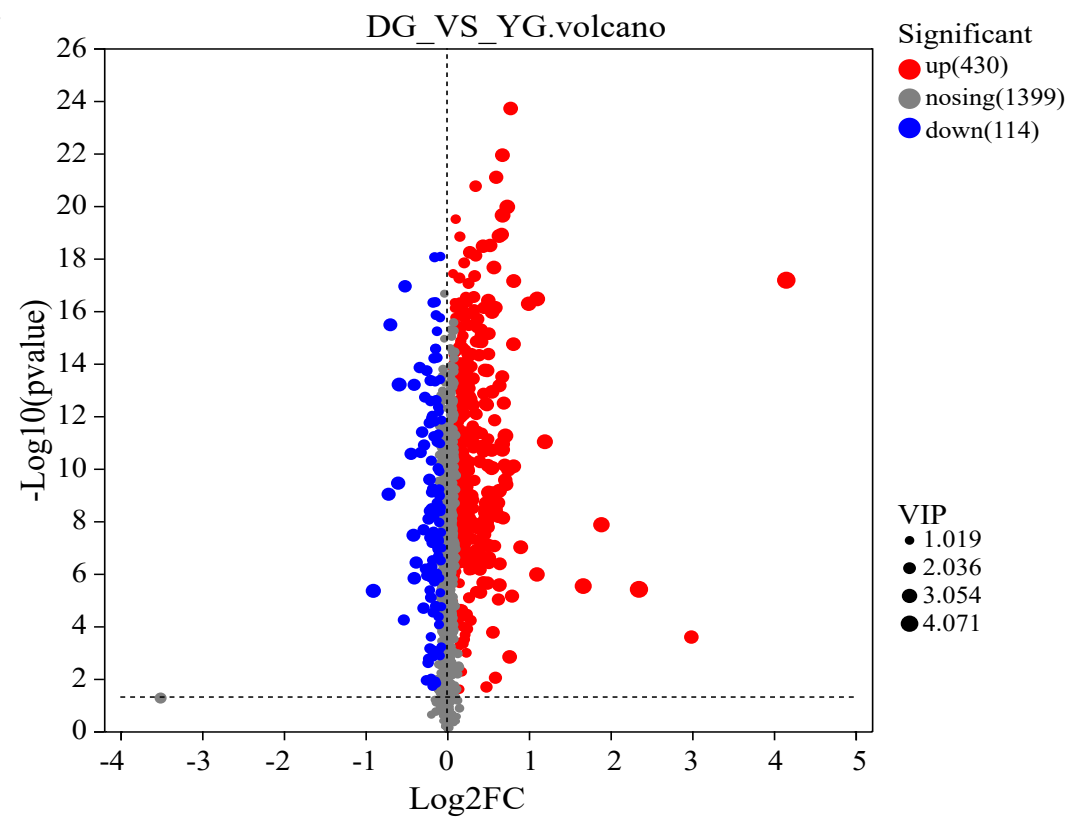

F

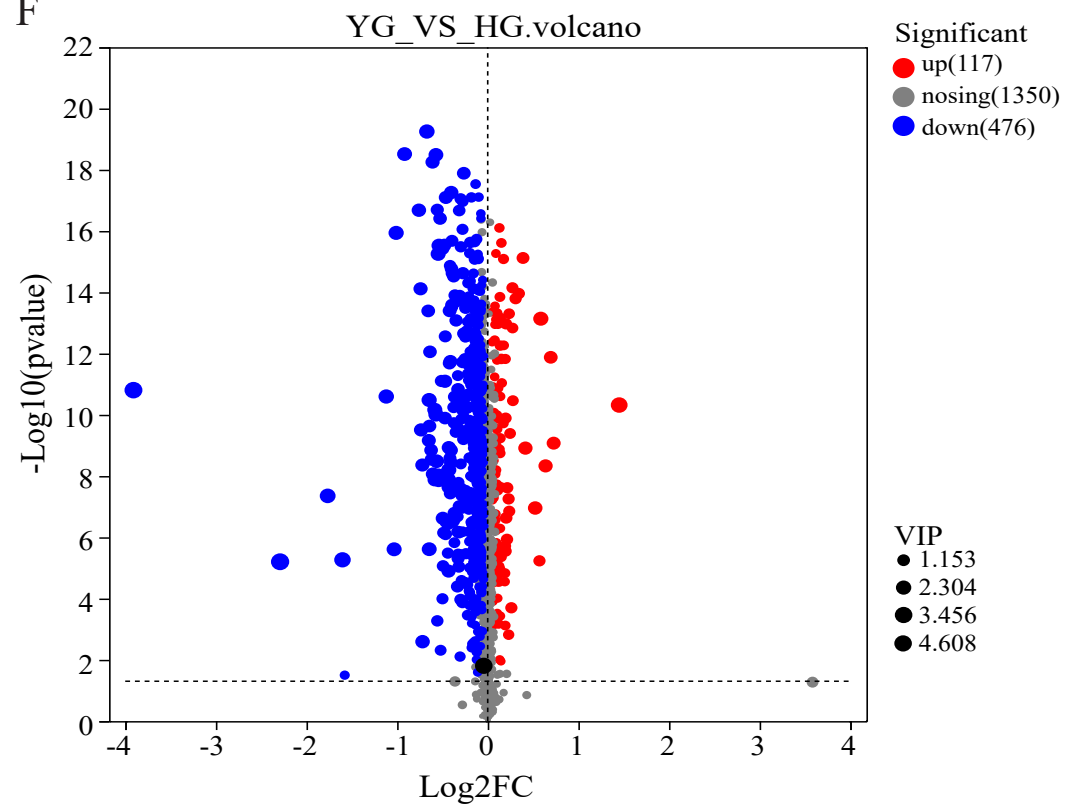

Supplement: Supplementary file 1 [file foods-13-01639-s001.zip › Figure S3.pdf]
